# Reliability and validity of a novel Kinect-based software program for measuring a single leg squat

**DOI:** 10.1186/s13102-020-00179-8

**Published:** 2020-05-11

**Authors:** John Ressman, Eva Rasmussen-Barr, Wilhelmus Johannes Andreas Grooten

**Affiliations:** 1 Department of Neurobiology, Care Sciences and Society, Division of Physiotherapy, Karolinska Institutet, Alfred Nobels Allé 23, 141 83 Huddinge, Sweden; 2 Allied Health Professionals Function, Functional Area Occupational Therapy and Physiotherapy, Karolinska University Hospital, Stockholm, Sweden

**Keywords:** Kinect, Movement screening, Single leg squat, Rehabilitation, Sports medicine

## Abstract

**Background:**

The Single leg squat (SLS) is a movement screening test widely used in clinical settings. The SLS is highly subjective in its nature. Objective measures, such as 3D-motion analyses, are seldom used in daily clinical work. An interactive, Kinect-based 3D-movement analysis system, the Qinematic™, is proposed to be easily used in clinical settings to assess the SLS. The aim of this study was to establish the test-retest reliability and construct validity of Qinematic™ for assessing the SLS. A further aim was to identify angles of medial knee displacement, to summarise the discriminative ability of the SLS measured by Qinematic™.

**Methods:**

We performed a test-retest reliability study (*n* = 37) of the SLS using Qinematic™ and a construct validity study, in which Qinematic™ data were compared with visual assessment of video-recorded SLS.

**Results:**

Three variables (left knee down, right knee up and down) reached “substantial reliability” (ICC = 0.64–0.69). One variable, “left knee up”, showed a significant difference between the two test occasions (T1–6.34°, T2 0.66°, *p* = 0.013, ICC = 0.50), and “poor absolute reliability” was seen for all variables (SEM = 9.04–10.66, SDC = 25.06–29.55). A moderate agreement between the visual assessment and Qinematic™ data for various knee angles was shown (Kappa = 0.45–0.58). The best discriminative ability of the SLS was found at a knee angle of 6° (AUC = 0.82, sensitivity = 0.86, specificity = 0.78, PPV = 0.58, NPV = 0.94).

**Conclusions:**

Qinematic™ shows a poor absolute reliability, and a substantial relative reliability, in measuring a SLS at the way down. This indicates that Qinematic™ should not be recommended for the use on an individual level, but it can possibly be used on a group level. The merged results of the construct validity study indicate that Qinematic™ at 6° of medial displacement can identify subjects with a knee over foot position. In summary, the use of the Qinematic™ net trajectory angle, which estimates the “line of best fit” cannot be recommended to assess a knee medial to foot position and should be reconsidered.

## Background

Visual assessment of movements is commonly used in the clinic to set and evaluate rehabilitation goals. In sports medicine, such tests aim to recognise the quality of movement, which is proposed to reveal a predisposition for injuries [1–4]. The Single Leg Squat (SLS) is a test that has gained widespread clinical use for assessing the movement quality of the lower extremity, and has also generated great research interest in its underlying biomechanics [5].

The SLS is performed by squatting from a single-leg stance, and is described, performed, and named in various ways, making it difficult to define a uniform test as “the SLS test” [6]. The SLS aims to measure the medial displacement of the knee, described as dynamic knee valgus, where the knee moves medial to the foot during a loaded position [7–9]. A dynamic knee valgus is characterised by an excessive pelvic drop, femoral internal rotation, knee valgus, tibia internal rotation and foot pronation [8, 9]. Some authors propose a simple approach, assessing only the relation between the foot and knee [10], whilst others propose a multi segmental approach, assessing the kinetic chain comprising the inter-relation between several body segments from the trunk to the foot [8, 9, 11]. In general, visual assessment is challenging as movements are complex and sometimes performed at high speed. Clinical examination is in addition subjective in its nature why it can be difficult for a test to reach acceptable levels of reliability and validity, thus hampering its clinical usage [12–14]. Reliability is commonly affected by the complexity of the rating scale, the definitions of the rating criteria, the velocity of the test, the between/within-subject variation, and the examiner’s training and clinical experience [6, 15, 16]. Even so, several studies report that visual assessment of the knee in relation to the foot is reliable and valid for asymptomatic adults [6, 10, 16, 17], whilst the multi segmental approach up until now has been questioned [16, 17]. A meta-analyses, however recently reported that a multi segmental approach, preferably with a ≤ 3 point rating scale, is indeed feasible and reliable [6]. The reason to assess more than one body segment is that additional information can be useful in the targeted rehabilitation [6, 16].

An increased dynamic knee valgus and its interlinked malalignment is proposed to be associated with overuse syndromes such as patella-femoral pain syndrome [18], iliotibial pain syndrome [19], femuro-acetabular impingement [20], tibial stress fractures [21] and injuries such as anterior cruciate ligament injuries [22]. No cut-off point is yet established for when the degree of a medial displacement of the knee is to be considered a risk for these syndromes or injuries. Further, there exists no precise or true consensus on the clinical importance using the SLS test to measure medial displacement of the knee. A critical step in injury prevention is to determine the cause of injury to understand why an athlete is at a greater risk in a given situation and how an injury occurs [23]. As the cause of injury often is multifactorial, comprehensive models to understand injury causations have been developed which emphasis intrinsic-, and extrinsic risk factors together with a careful description of the injury [23, 24]. Intrinsic factors are among others described as modifiable physiological factors [7, 23, 25] such as the dynamic knee valgus, visually assessed with for example the SLS test, drop jump, vertical drop jump, lunge, one leg hop or a crossover hop [16, 17]. In addition to separate movement tests, a variety of screening systems are used in the clinic to observe and assess injured athletes or for proactive purposes among others the Functional Movement Screen (FMS) [1, 2, 26, 27].

The use of objective and quantitative reliable measures to study movements measures are not accessible for all clinicians, and might be impractical, as they are time consuming and not applicable in larger populations. Unfortunately, these measures are mostly available in laboratory settings [28]. In 2010, Microsoft released Kinect for Xbox 360 as a game controller aiming to capture 3D movements of the human body via the data acquired from a built-in RGB sensor and a skeleton-tracking algorithm from the Kinetic Software Development Kit (SDK) [29, 30]. This portable, marker-free, and low-cost sensor has been used and evaluated in various diagnoses; in movement of people with Parkinson’s disease [31], in standing balance [30, 32] and in movements of upper and lower extremities [33, 34]. For movement of the lower extremity, the test-retest reliability, accuracy, and construct validity for different functional tests, such as the SLS, has been investigated [34–39]. Qinematic™ is a novel, interactive, motion analysis system that uses the Kinect camera together with a refined software program (Quickposture™) that has improved the camera’s stability and accuracy by using a unique tracking algorithm [40]. It works as a semi-automated service that records, measures, and reports movements, and is intended to be used by health- and wellness service providers in workplaces, gyms, and clinics [40]. Seemingly, Qinematic™ might be an alternative to visually assessing movement quality and might thus be an important step in future health care digitisation. However, before Qinematic™ can be suggested for use in clinical practice or research, it is important to establish reliability and validity [41]. Grooten et al. [42] previously investigated the psychometric properties of Qinematic™ and showed poor validity and reliability for the test’s ability to measure balance, posture, and side-bending. The dynamic tasks of Qinematic™, such as single and double leg squat, were not investigated and still require further research.

Albeit a highly significant correlation between dynamic knee valgus and injury risk is reported, it still is not possible to predict future injuries based on the results from movement screening tests [43]. The need to improve and develop methods and the understanding for the complexity of injury prevention and thus movement screening, still remains an important and essential part of the effort to protect the athlete from injury [44]. Hence, there is a need for simpler, yet objective and quantitative, methods for capturing dynamic knee valgus. Such methods could be used to define relevant cut-offs, or angles associated with a greater risk of knee injury, when evaluating movement quality with a SLS.

The aim of the present study was therefore to establish the test-retest reliability (relative and absolute) and the construct validity of Qinematic™ for assessing the SLS. A further aim was to identify angles or cut-off points of medial knee displacement, during a SLS measured by Qinematic™, that in the best way would match the results of a visually assessed knee over foot or knee medial to foot position.

## Methods

### Design

A study on the test-retest reliability of the SLS using Qinematic™ was conducted by having subjects perform one session of Qinematic™ on two different occasions, six to seven days apart [45]. Afterwards, the construct validity of Qinematic™ was studied by comparing the data obtained from Qinematic™ with a video-recorded visual assessment of the SLS conducted by two experienced physiotherapists. Several angles, or cut-off points, of medial knee displacement from the Qinematic™ data were used to compare the visually assessed knee over or medial to foot position.

### Subjects

Thirty-seven healthy and active persons (27 women, 10 men) were recruited via verbal announcement and information posters at the Karolinska Institutet in Stockholm. Inclusion criteria were men and women, aged 18 to 65. Exclusion criteria were an ongoing musculoskeletal injury in the lower extremity, a history of serious knee disorder (ligament- or meniscal rupture and knee replacement), a neurological disease, or a visual deficiency that couldn’t be corrected with glasses. The test subjects’ characteristics, pain, and activity levels are described in Table 1.

**Table 1:** Test subjects’ characteristics, pain, and activity levels

	All (***n*** = 37)	Women (***n*** = 27)	Men (***n*** = 10)
Age, year Mean (SD)	34 (12)	34 (12)	34 (10)
Height, cm Mean (SD)	173 (7)	169 (5)	181 (5)
Weight, kg Mean (SD)	70 (14)	65 (8)	86 (14)
Physical active ≥2 days/week^a^ % of group (n)	81% (30)	82% (22)	70% (7)
Pain in regions other than the lower limb % of group (n)	27% (10)	26% (7)	30% (3)

^a^Most common physical activities: running/jogging and weightlifting, but yoga, swimming, power walks and cycling were also reported

### Data collection

**Fig. 1:**
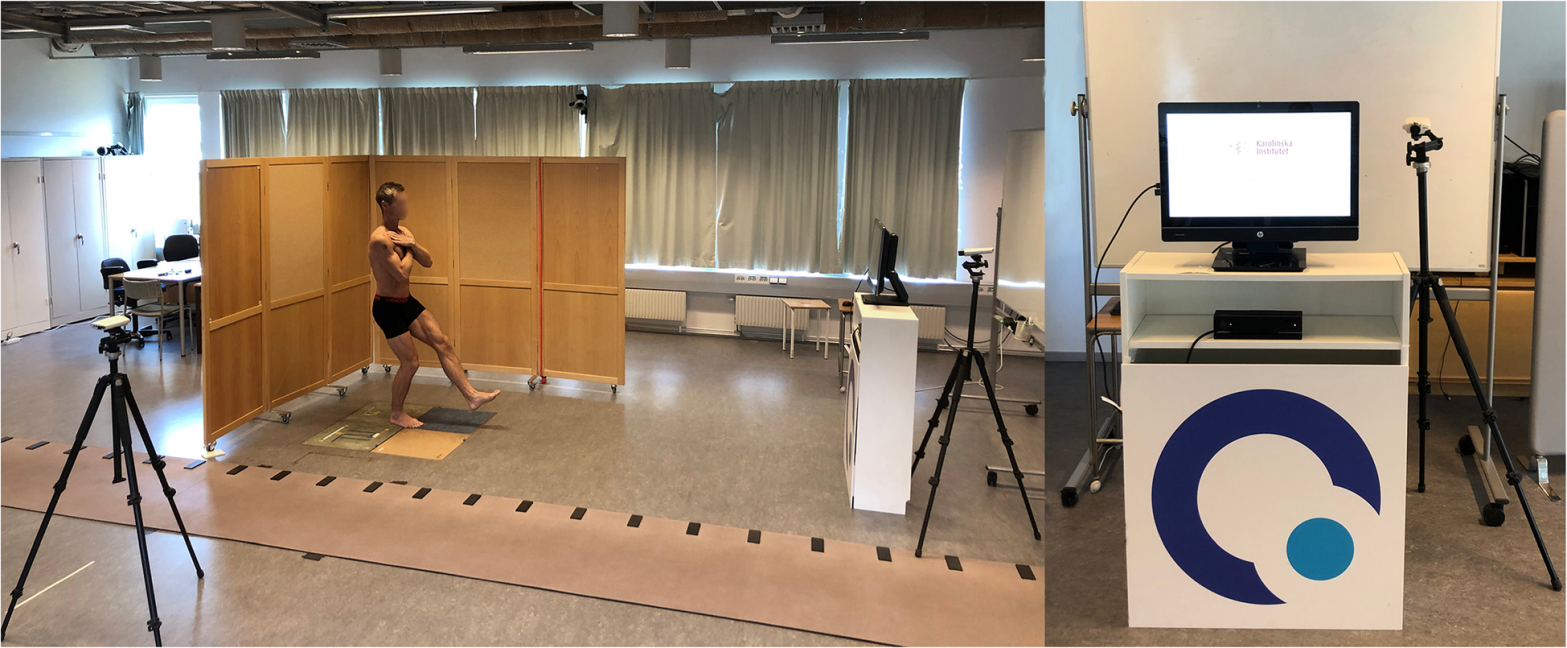
The set-up of Qinematic™ during the data collection of the Single Leg Squat. The two orthogonally placed cameras are shown in the figures, right figure shows the subjects view in front of the Kinect camera and computer touchscreen

Before the tests, all subjects filled in a questionnaire concerning their demographics and background data and gave their informed consent. All tests were performed at the movement laboratory of the Karolinska Institutet during 21 March and 11 May 2017. The tests were administrated by two of the authors (JR and WG). The subjects were instructed to wear shorts/tights and a singlet. The tests in front of the Qinematic™ system and digital video cameras had a duration of approximately 10 min. The subjects received oral and visual instructions while standing in front of the Kinetic camera of the Qinematic™ system, which incorporates a computer touchscreen (size 23 in.) that was placed on a specially constructed cabinet. The Posture Scan software asks the person to stand at a calculated and relative distance from the Kinect camera that is suitable for the subject’s height in order to control for the height differences between people. The Kinect camera was placed in the same cabinet under the computer screen at a height of 82–86 cm (Fig. 1). Simultaneously with the Qinematic™ session, two orthogonally placed digital video cameras (Axis Communications 210A) recorded all trials at 100 Hz in the sagittal and frontal plane at three meters’ distance, and these cameras were placed so that the whole body was visible with a brown even background. Ethical approval (Dnr: 2016/595–31 with amendment Dnr 2017/318–32) was obtained on 2016-03-09 and 2017-01-19, respectively.

#### Qinematic™-procedure

The standard Qinematic™ movement screening test includes seven different functional tasks that are performed in the following order: #1. Standing balance; #2. Side bending; #3. Squat (double leg squat) with arms crossed over the chest; #4. Balance on the right leg with arms crossed over the chest; #5. Balance on the left leg with arms crossed over the chest; #6. Squat on the right leg (SLS) with arms crossed over the chest; and #7. Squat on the left leg (SLS) with arms crossed over the chest. For the purpose of the present study, only data from #6 and #7 (SLS) were presented. A further description of Qinematic™ test procedure and test instructions is reported elsewhere [40, 42]. This study used Posture Scan version 2.1.14 (Qinematic AB, Sweden), Movement Lab version 2.1.20 (Qinematic AB, Sweden), a Hewlett Packard computer with Windows 10 Pro software and a Microsoft Kinect sensor (v2), which samples data at a frequency of 30 Hz, to collect the data. The specific instructions for the SLS test left leg were: “stand on your left leg and lift your right leg in front of you. Bend your left knee and rise up again”. Corresponding instructions were given for the right leg. Qinematic™ has no prespecified depth of knee flexion to be achieved during the SLS, but the non-weight bearing leg is not allowed to cross the midline. If a movement was not properly conducted due to a misunderstanding of the instructions, a wrong positioning of the body parts, or a loss of balance, Qinematic™ detected thisas a “no-go” and the procedure was repeated, at a maximum of three times. If Qinematic™ was still not satisfied, the subjects were asked to perform an easy form (“easy mode”) of the SLS, with the heel of the non-tested leg placed on the ground in front of the tested person as a support. If Qinematic™ was still not satisfied with the performance, this movement was recorded as a missing value.

#### Visual assessment-procedure

For the construct validity study, two raters (JR, ERB) visually assessed the SLS video recordings that were recorded in parallel to the Qinematic™ procedure. The rating of the SLS was dichotomized as the subject having a knee over foot position (pass), or a knee medial to foot position (fail). A knee over foot position was considered as a pass, when the knee was well aligned over or lateral to the second toe. A knee medial to the foot position was scored as a fail, when the knee was well medial to the second toe [10].

The subjects were recorded in the sagittal and frontal plane at the same time. The raters’ were instructed to study the video recordings at a maximum of three times without pausing or using slow-motion function. The rating criteria were discussed among the raters’ to reach a consensus on how to assess the tests before the assessment of the video recordings started. Eleven video recordings, across all trials, were randomly chosen and individually assessed to reach the consensus in how to assess. Following this, all video recordings, were individually assessed by two raters’ (JR and ERB). Consensus was reached in all cases without the need to consult the third author (WG).

### Variables and data management

**Fig. 2:**
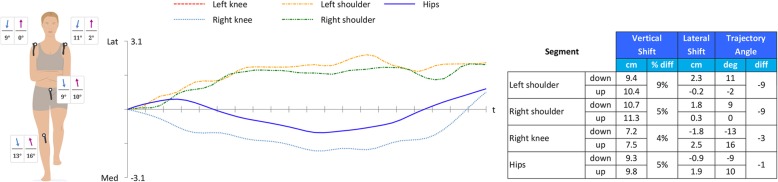
The biomechanical report of Qinematic™ for the SLS. The net trajectory angle (NTA) estimates the “line of best fit” for the pathway of different key body parts, the table shows 13° of medial displacement for the right knee on the way down, and 16° of lateral displacement on the way up, but only 1.8 cm and 2.5 cm of medial/latera shift, respectively, blue dotted line in the chart

Qinematic™ provides different variables for the movement screening tests in their biomechanical reports (Additional file 1). For the SLS, three variables are reported (shoulder, hip, and knee), but in the present study only the variable of the knee was of interest. Qinematic™ calculates a net trajectory angle (NTA) that estimates the “line of best fit” for different key body parts, which for the SLS and the knee are all lateral/medial movements in the frontal plane against the vertical axis, on the way up and down, see Fig. 2. All tangents are measured 30 times per second from the top of the squat to the bottom of the squat and vice versa after the turning point. The data provided by the Qinematic™ software were used for both the reliability and the validity studies. For the validity study, the SLS data on the “way down”, and not the “way up”, were used in the comparison with the visual assessment. The reason for this was that the “way up” showed poor reliability with a significant difference between the test occasions in the reliability study. In both the reliability and the validity part of the study, data was excluded in the cases with missing values, or if the test person performed an easy form (“easy mode”) of the SLS on one or both occasions. Qinematic™ data were used continuously in the reliability study. To find the optimal limits for construct validity, data were dichotomized (fail/pass) in steps of two degrees, up to 20 degrees of medial displacement of the knee. The medial displacements, measured in degrees by Qinematic™, was then compared to the dichotomized (fail/pass) visual assessment of the knee position in relation to the foot as described above.

To check for normality, means and medians were compared, together with visual analyses of histograms and distributional diagnostic plots. We also tested for skewness and kurtosis [46]. Two out of eight variables showed non-normally distributed data (left knee down and right knee up at occasion one) and in order not to overestimate our data, medians, interquartile range (IQR) and non-parametric statistics were used for the descriptive statistics.

### Statistics

#### Test-retest reliability

The median and interquartile range (IQR), in degrees of medial displacement of the knee, was calculated for the “way down” and the “way up” for both legs and separately for test occasions one and two. The grand median was calculated for both test occasions. Wilcoxon signed-rank test was used to test for the occurrence of a systematic difference between the two test occasions. The level of significance was set to *p* < 0.05.

Relative reliability was calculated using the Intraclass Correlation Coefficient (ICC 3.1), where a two-way mixed effect model, absolute agreement, single rater/measures was used [47, 48]. The ICC-levels were classified as: < 0.00 = poor; 0.00–0.20 = slight; 0.21–0.40 = fair; 0.41–0.60 = moderate; 0.61–0.80 = substantial; and 0.81–1.0 = almost perfect [49, 50].

Since not all variables were normally distributed, Spearman correlation coefficients were calculated in addition to the ICCs. The Spearman correlation coefficient was interpreted as: less than 0.3 low correlation, 0.3–0.5 fair correlation, 0.6–0.8 moderately strong correlation, at least 0.8 very strong [51, 52].

Absolute reliability was calculated via the Standard Error of Measurement (SEM) and Smallest Detectable Change (SDC). These measures express the measurement error in the same unit as the original measurement for use on an individual level [47, 53]. The SEM is a measure of how far apart the outcomes of repeated measurements are, what the standard deviation around a single measurement is; while the SDC is a change beyond measurement error, where a change in the construct can be considered real [47, 53]. As the parameters of SEM and SDC are expressed in the unit of measurement, they should be as low as possible in this study. Another term used for SDC is Minimal Detectable Change (MDC), which will be used synonymously within this text. Finally, we plotted Bland-Altman plots in order to detect any systematic differences or proportional bias (Additional file 2) [47, 54].

#### Construct validity

Agreement of the visually assessed SLS and the measures of Qinematic™ for each cut-off point, was calculated by percent agreement (PA) and Kappa statistics, with a 95% confidence interval (95% CI). Diagnostic accuracy, defined as the amount of agreement between the index test (Qinematic™ for each tested cut-off point) compared to the reference standard (the visual assessment of the SLS) [55, 56], was assessed by calculating the area under the receiver operation characteristic (ROC) curve, together with the standard error (SE) and the 95% CI.

A ROC curve is a plot of sensitivity against specificity, and was calculated for all possible cut-off points, as the ROC curve essentially gives a single measurement that summarises the discriminative ability of a test at that specific cut-off point [57]. An index of the “goodness” of the test is the area under the curve (AUC), and a perfect test yields an AUC of 1.0. As a rule of thumb, the following classification has been suggested: > 0.9 = high accuracy; 0.7–0.9 = moderate accuracy; 0.5–0.7 = low accuracy; and 0.5 = a chance result [45, 57]. Finally, positive predictive value (PPV) and negative predictive value (NPV) were calculated to investigate the probability that a subject had a knee over foot position, or a knee medial to foot position, when Qinematic™ exceeded or did not exceed a given cut-off point. STATA version 15.1 was used to run all statistical analyses and Microsoft Office Excel version 16 for Windows 10 was used to plot the Bland-Altman plots.

## Results

### Subjects

Thirty-seven subjects participated in the reliability and validity studies. No one dropped out of the study. For the reliability study the 37 subjects all together produced 296 Qinematic™ measures, as both legs were measured during both the “way up” and the “way down” on both test occasions. In summary, 85% of the data were available for the test-retest reliability study.

Thirty-two of the measures were excluded due to that the test person either performed an “easy mode” on the first or second test occasion (28/32) or an “easy mode” on both occasions (4/32). Ten of the measures were classified as missing data due to Qinematic™ not reporting any values on the first or second occasion. In addition, one measure was assessed as improper by the authors of the study.

For the validity study, 37 subjects produced 148 video recordings. In total, 76% of the data were available for the validity study. Fifteen of those were excluded due to that the test person performed an “easy mode”. Missing data were classified due to; poor video quality (4/20), the tested person losing their balance (2/20) and missing video recordings (13/20). For one test person two SLSs were video recorded, but only one was recorded by Qinematic™ (1/20).

### Test-retest reliability

All psychometric data from the test-retest data are presented in Table 2. Since two variables were not-normally distributed (left knee down and right knee up at occasion one), Spearman correlation coefficients were calculated in addition to ICCs to assess relative reliability. Three variables (*left knee down, right knee up, and right knee down*) reached “substantial reliability”, with ICC’s ranging from 0.64 to 0.69. These variables did not differ statistically between the two occasions (*p* = 0.21). For the same three variables, Spearman correlation coefficient reached “moderately strong” (r=0.61-0.68).

**Table 2:** Results from the test-retest reliability study

	Data^a^			Differences^b^	Relative reliability^c^		Absolute reliability		
	T1 Median (Q1, Q3)	T2 Median (Q1, Q3)	GRAND Median (Q1, Q3)	T1 vs. T2 p-value	Spearman (r) *p*-value	ICC (3.1) (95% CI)	SEM^d^	SDC^e^	Mean difference between T2 and T1 with 95% confidence interval (95% CI)^f^
**Left knee up** (*n* = 64)	−6.34 (−16.93, 6.26)	0.66 (−8.40, 10.63)	−2.53 (−11.53, 9.20)	0.013*	0.53 (0.002)	0.50 (0.17 to 0 .72)	10.66	29.55	6.89 (1.98 to 11.81) *
**Left knee down (**n = 64)	1.07 (−6.50, 19.42)	0.80 (−9.50, 13.42)	1.07 (−6.76, 15.48)	0.5496	0.68 (< 0.001)	0.69 (0.45 to 0.83)	9.85	27.30	−2.15 (−7.19 to 2.89)
**Right knee up** (*n* = 61)	−6.48 (−16.59, 7.09)	−1.95 (−10.35, 7.87)	−3.20 (−12.06, 7.09)	0.2059	0.61 (< 0.001)	0.69 (0.45 to 0.84)	9.04	25.06	2.83 (−1.91 to 7.56)
**Right knee down (***n* = 62)	0.59 (−11.71, 13.52)	2.75 (−10.04, 12.59)	2.27 (−10.04, 12.81)	0.5967	0.61 (< 0.001)	0.64 (0.38 to 0.81)	9.09	25.20	1.24 (−3.53 to 6.00)

*Denotes a statistically significant change; *n* = denotes the number of measurements done by Qinematic™ for each variable; A negative value (−) denotes a medial displacement for the left knee and a lateral displacement for the right knee, contrariwise a positive value (+) denotes a medial displacement of the right knee and a lateral displacement for the left knee

^a^ Test occasion 1 = T1, Test occasion 2 = T2, Q1: 1st quartile (25%), Q3: 3rd (75%). Grand Median = median of all measures from test occasion 1 and 2. All data are in degrees

^b^ Wilcoxon signed-rank test (paired test). A *p*-value of *p* < 0.05 was considered to be statistically significant and marked with*

^c^ Reliability based on a two-way mixed effect model, calculating the absolute agreement, based on single ratings. ICC (3.1):${ICC}_{agreement}=\frac{\sigma_p^2}{\sigma_p^2+{\sigma}_o^2+{\sigma}_{residual}^2},{\sigma}_{error}^2={\sigma}_o^2+{\sigma}_{residual}^2$

^d^ SEM: Standard error of the measurement: ${SEM}_{agreement}=\surd \left({\sigma}_o^2+{\sigma}_{residual}^2\right)$

^e^ SDC: Smallest detectable change: *SDC* =  ± 1.96 ∗  √ 2 ∗ *SEM*_*agreement*._

^f^ Mean difference between the two-test occasion was calculated together with the 95% confidence interval (95% CI). *A 95% CI that does not include zero indicates a systematic change in the mean between T1 and T2.

The variable “*left knee up*” showed a significant difference on the two occasions (T1–6.34°, T2 0.66°, *p* = 0.013), and showed a “moderate reliability” of ICC 0.50 (CI 95% 0.17–0.72). For the same variable, Spearman correlation coefficient reached a “fair correlation” (r 0.53). The absolute reliability measures of SEM and SDC were relatively high for all four variables, indicating that they are less appropriate for use on an individual level (Table 2). The Bland-Altman plots (Additional file 2) were visually analysed and confirmed a systematic difference for the variable “*left knee up*”, but no other systematic differences or proportional bias was found.

### Construct validity

The proportion of subjects rated as a “knee medial to foot position” in the investigated population was 30 out of 113 possible SLS, giving a prevalence of 27%. For Qinematic™, this proportion varied between 46 and 12%, when using 2° and 20° as cut-off points, respectively. At 6°, the prevalence of “fails” found by Qinematic™ was 39%.

Psychometric data on the construct validity are presented in Table 3.

**Table 3:** Results from the construct validity study

	Agreement^b^		Diagnostic accuracy		Predictive values*		ROC^**i**^
Cut off scores for index test^a^	PA^**c**^	Kappa^**d**^ (95% CI)	Sensitivity^**e**^ (95% CI)	Specificity^**f**^ (95% CI)	PPV^**g**^ (95% CI)	NPV^**h**^ (95% CI)	AUC^**j**^ (SE, 95% CI)
Qinematic™ 2°	0.75	0.47 (0.30 to 0.64)	0.90 (0.73 to 0.98)	0.69 (0.58 to 0.79)	0.51 (0.42 to 0.60)	0.95 (0.86 to 0.98)	0.79 (0.04, 0.72 to 0.87)
Qinematic™ 4°	0.77	0.51 (0.34 to 0.68)	0.86 (0.68 to 0.96)	0.74 (0.63 to 0.83)	0.54 (0.45 to 0.64)	0.94 (0.86 to 0.97)	0.80 (0.04, 0.72 to 0.88)
**Qinematic™ 6°**	**0.80**	**0.55 (0.39 to 0.73)**	**0.86 (0.68 to 0.96)**	**0.78 (0.67 to 0.86)**	**0.58 (0.47 to 0.68)**	**0.94 (0.86 to 0.98)**	**0.82 (0.04, 0.74 to 0.90)**
Qinematic™ 8°	0.83	0.57 (0.39 to 0.75)	0.72 (0.53 to 0.87)	0.86 (0.77 to 0.93)	0.66 (0.51 to 0.78)	0.90 (0.83 to 0.94)	0.79 (0.05, 0.70 to 0.89)
Qinematic™ 10°	0.84	0.57 (0.39 to 0.75)	0.66 (0.46 to 0.82)	0.90 (0.82 to 0.96)	0.70 (0.54 to 0.83)	0.88 (0.82 to 0.92)	0.78 (0.05, 0.68 to 0.87)
Qinematic™ 12°	0.82	0.50 (0.30 to 0.70)	0.55 (0.36 to 0.73)	0.91 (0.83 to 0.97)	0.70 (0.51 to 0.83)	0.85 (0.79 to 0.90)	0.73 (0.05, 0.65 to 0.83)
Qinematic™ 14°	0.85	0.58 (0.39 to 0.77)	55.17 (0.36 to 0.74)	0.96 (0.90 to 0.99)	0.84 (0.63 to 0.94)	0.86 (0.80 to 0.90)	0.76 (0.05, 0.66 to 0.85)
Qinematic™ 16°	0.86	0.57 (0.37 to 0.76)	0.52 (0.33 to 0.71)	0.98 (0.91 to 1.0)	0.88 (0.65 to 0.97)	0.85 (0.80 to 0.89)	0.75 (0.05, 0.65 to 0.84)
Qinematic™ 18°	0.85	0.54 (0.33 to 0.74)	0.48 (0.30 to 0.68)	0.98 (0.91 to 1.0)	0.88 (0.63 to 0.97)	0.84 (0.79 to 0.88)	0.73 (0.05, 0.64 to 0.82)
Qinematic™ 20°	0.83	0.45 (0.23 to 0.68)	0.38 (0.21 to 0.58)	0.99 (0.93 to 1.00)	0.92 (0.60 to 0.99)	0.82 (0.77 to 0.86)	0.68 (0.05, 0.59 to 0.77)

*Abbreviations*: *95% CI* 95% Confidence interval, *SE* Standard error

^a^Cut-of scores for index test Qinematic™: Qinematic™ 2° = Knee goes 2 degrees medial at the way down during a Single Leg Squat

^b^Agreement: Agreement of the visual assessed Single Leg Squat and the measures of Qinematic™ at different cut-off scores for medial displacement of the knee

^c^PA: Percent agreement

^d^Kappa: Cohens’ kappa, calculated by; $\mathrm{K}=\frac{P_0-{P}_e}{1-{P}_e}$

^e^Sensitivity: Probability that Qinematic™ exceeds the given cut-of score when the subjects are assessed as having a knee-medial-to-foot position, a true positive rate

^f^Specificity: Probability that Qinematic™ doesn’t exceed the given cut-of score when the subjects are assessed as having a knee over-foot position, a true negative rate

^g^Positive predictive value: Probability that the subjects are assessed as having a knee-medial-to-foot position when Qinematic™ exceeds the given cut-of score. *The prevalence of having a “knee-medial-to-foot position” in the investigated population are 27%

^h^Negative predictive value: Probability that the subjects are assessed as having a knee-over-foot position when Qinematic™ doesn’t exceed the given cut of score. *The prevalence of having a “knee-medial-to-foot position” in the investigated population are 27%

^i^ROC Receiver Operating Characteristics in which the true positive value (sensitivity on Y-axis) is plotted against the false positive value (1-specificity on X-axis)

^j^AUC Area Under the Curve

Regarding the agreement of the visually assessed SLS and the measures of Qinematic™, Kappa statistics ranged from 0.45 (20° cut-off) to 0.58 (14° cut-off), which indicates a “moderate agreement”. All in all, the percent agreement ranged from 75% (2° cut-off) to 86% (16° cut-off). Logically, the sensitivity was highest when using a cut-off point at 2° of medial displacement, 0.90 (95% CI 0.73 to 0.98), and lowest when using a cut-off at 20°, 0.38 (95% CI 0.21 to 0.58). For specificity, the relationship was reversed for these cut-off points, and ranged between 0.99 (95% CI 0.93 to 1.0) at a cut-off at 20°, and 0.69 (95% CI 0.58 to 0.79) at a cut-off at 2°. The highest area under the curve (AUC) was reported when using a cut-off at 6° of medial displacement, and showed a measure of 0.82 (SE 0.04, 95% CI 0.74–0.90), which indicates a “moderate” accuracy [45, 57]. At the same cut-off point, the PPV was 0.58 (95% CI 0.47 to 0.68), and the NPV was 0.94 (95% CI 0.86 to 0.98).

## Discussion

This study explored the test-retest reliability and construct validity of a novel software program, Qinematic™, in assessing the SLS, and found a “substantial” relative reliability for the way down, but not for the way up. However, the absolute reliability showed large SEM and SDC for both the “way up” and the “way down”. A further aim was to identify the most appropriate knee angle of medial displacement, and here a “moderate” agreement was found between the visually assessed SLS and the measures of Qinematic™ for all different cut-off points. The best AUC was found at a cut-off point of 6°, indicating a “moderate” accuracy, and for the same cut-off point, the PPV and NPV measured 0.58 (95% CI 0.47 to 0.68) and 0.94 (95% CI 0.86 to 0.98), respectively.

To our knowledge, few studies have so far investigated the test-retest reliability or construct validity of the Qinematic™ software program in measuring the SLS. Grooten et al. [42] recently investigated the psychometric properties of Qinematic™ in various tests and reported poor validity and reliability for the ability of Qinematic™ to measure balance, posture and side-bending. Unfortunately, the results from recent studies that used the Kinect camera and investigated the SLS and the double leg squat cannot be compared with the Qinematic™, since different post-processing techniques are used to measure lower extremity kinematics [34–37, 39]. While, most studies measure peak joint angles [34–37, 39], Qinematic™ calculates the medial and the lateral displacements of the knee 30 times per second from the top of the squat to the bottom of the squat (down) and vice versa (up). Then, a net trajectory angle (NTA) is calculated which represents the angle between the estimated “line of best fit” through the changes in knee position and the vertical axes for each direction.

The present study found a systematic difference between test occasions one and two for the way up for the left leg, and therefore this movement was not used in the validity study. To be noted, there was no difference between the test occasions for the right leg. This indicates that the difference noted between the legs might depend on the fact that the test persons always started with their left leg, and that there could have been a motor learning effect that possibly could have influenced (stabilized) the performance of the right leg.

Previous test-retest studies [36, 37, 39] using Kinect data present relatively small and clinical acceptable SEM and MDC values. Mentiplay et al. [36] measured the knee abduction angle and calculated the SEM of a SLS to 4.38° and 3.62° for a vertical drop jump. Similar low values were found by Schmitz et al. [37] in which an MDC of 3.1° were found for knee adduction and 4.1° for knee flexion in a double leg squat. Wochatz et al. [39] found, however, somewhat higher variation for knee flexion/extension (SEM = 6.8–8.3°) during a double leg squat. They concluded that the Kinect V2 system could reliably assess lower limb joint angles and positions during simple movements, but that the reliability decreased with increasing complexity of the movement (this is in order: Double Leg Squat-Hip abduction-Lunge), and that discrepancies occurred in the detection of joint angles and positions with small movement amplitudes. Grooten et al. [42] reasoned that one cause for the poor reliability and validity found in measuring posture, balance and side bending with the Qinematic™ system might have been the large individual variation in performing the tests between the occasions. In contrast, kinematic test-retest studies for the knee and hip during a SLS, or step-down manoeuvres, show SEM and MDC values in the range of 1.3 to 8.3 degrees [36, 37, 39, 58–61]. This relatively small within subject- and between days kinematic variation for the SLS and step-down manoeuvres indicate a clinically acceptably absolute reliability, which is the most important type of reliability for a clinician to consider when assessing performance or making clinical decisions on an individual level. In this aspect, Qinematic™ showed far too high SEM and SDC values to be used in clinical settings for monitoring individuals from time to time. When analysing the video recordings, it was clear that the high SEM and SDC values were not a result of a large within-subject variation in knee abduction angles during their performance of the SLS. Instead, this may be an effect of the small medial and lateral displacements of the knee occurring during the SLS, resulting in large angles of the net trajectory angles (NTA) which estimate the “line of best fit” during the whole movement. Although the idea of using the NTA and not only the maximal knee displacement angle might be of value, the use of NTA and the “line of the best fit” cannot be recommended for the use to assess a knee medial to foot position, and should therefore be reconsidered.

Schmitz et al. [37] showed better test-retest reliability compared to Wochatz et al. [39] regarding the performance of a double leg squat, and proposed that this was due to a better standardization of the test performance with restricted knee flexion and controlled movement velocity. This might also be the case for the Qinematic™ as the depth and velocity of the SLS are not specified, even though the Qinematic™ by itself does correct an improper SLS. Furthermore, looking at the screen while listening to instructions and at the same time performing a SLS, might have been too much input at one time, and an additional reason for the higher SEM and SDC values. A dual task interferes with motor performance [62]. On the other hand, Qinematic™ showed better results on a group level, where the relative reliability was classified as “substantial” for the way down of a SLS (ICC = 0.64/0.69). This indicates that Qinematic™ might be used to monitor a group of for example athletes over time. In terms of reliability, recent studies highlight difficulties with the hardware as well as the software of the Qinematic™ system when measuring knee angles in the frontal plane during concurrent internal and external rotations of femur and tibia during a SLS [36, 37, 39]. Moreover, the post-processing algorithms of Qinematic™ (the NTA) which enables capture of the whole movement in one point, seems to be more unreliable compared to the attempts of capturing peak angles at one specific point during the movement.

Wochatz et al. [39] concluded that there is an indication for further development of advanced software and real-time post-processing techniques that improve the precision and validity of the Kinect V2 system, as compared to 3D laboratory equipment. This concurs with other studies that attribute a good validity of the Kinect to their customized processing techniques [36, 37]. Construct validity should be used when a gold standard is lacking, and assumes that the measurement instrument validly measures the construct to be measured [47, 63]. In the present study, we used visual assessment of the SLS as a construct to validate the Qinematic™ as previous kinematic studies have shown good validity for visual assessment of the SLS [10, 64–67]. Our results showed a “moderate” agreement between the visually assessed SLS and the Qinematic™ for all the different cut-off points. The best diagnostic accuracy was found for a cut-off point of 6° at “the way down”, indicating a “moderate” accuracy. At this cut-off point, the sensitivity was calculated to 0.86 and the specificity to 0.78. This means that a subject having a knee medial to foot position has an 86% probability of being classified as such, and that the corresponding probability of being classified as having a knee over foot position is 78%. Although these results seem to be promising, one should keep in mind that there could be a 14% (sensitivity) and 22% (specificity) misclassification on a group level. On the other hand, in a clinical perspective, additional diagnostic parameters, such as the predictive values might be more informative compared to sensitivity and specificity. In the present study, an NPV of 0.94 was shown at the cut-off point of 6°. This demonstrates that the subjects who did not exceed 6° of medial displacement had a knee over foot position, and that very few were classified as false-negative. In this perspective, Qinematic™ might be used to exclude subjects from further examination as having a knee over foot position when the medial displacement of the knee is less than 6° on the way down performing a SLS. On the contrary, the PPV at the cut-off point of 6° was calculated to 0.58, which implies that the probability of a subject being assessed as having a knee medial to foot position when the knee exceeded 6° of medial displacement was 58%, but also that 42% of those exceeding 6° were false-positive, which is a too high proportion of misclassifications to make conclusions on movement quality. Moreover, the 12% difference in prevalence of knee medial to foot position measured by Qinematic™ and the video-recorded visual assessment at 6° indicates that Qinematic™ classifies too many false-positives. On the other hand, a false-positive misclassification of a SLS would not lead to any greater harm, even if a treatment was initiated for those without poor movement quality, which could justify a lower PPV. Considering all diagnostic accuracy measures and predictive values at the cut-off point of 6°, the merged results show that Qinematic™ is good at identifying subjects with a knee *over* foot position, but for those showing a knee *medial* to the foot position might need to be assessed with a an additional test; perhaps a vertical drop jump [68] or a similar test that places a higher demand on the knee.

From a clinical perspective, it can be debated if the use of Qinematic™ adds any new information as clinicians apparently are able to visually assess a knee over foot position with a good accuracy. A valuable use of the Qinematic™, in the sense of knee assessment might be as a pedagogic tool in dialog with the patient [35].

### Methodological considerations

One strength of the present study is the methodological structure, in which the COnsensus-based Standards for the selection of health Measurement INstruments (COSMIN) [41, 69], and the Quality Assessment of Diagnostic Accuracy Studies (QUADAS) [70] were used. For validity and reliability studies, a sample size with at least 50 measures is recommended [47, 71]. This study enrolled 37 subjects, and assessed both the left and right legs, which gave around 74 separate measures. This can be seen as an appropriate amount of data fulfilling the requirement of at least 50 data points. Although one could argue that data provided by two legs of one subject are not independent data, the presence of differences between the legs, i.e. leg dominance (balance/strength), side differences in ankle mobility/calf flexibility, together with previous injuries, could justify the use of the data as different and independent measures. The study population was a convenience sample of both men (27%) and women (73%), with an average age of 34 years (SD 12), who were relatively active. This might be seen as one target group for Qinematic™, but no further generalisation to another population can be made from our findings. Furthermore, as the PPV and NPV are directly related to the prevalence of the “disease” in the population, our values could have been different with a higher prevalence of knee medial to foot position (27% in this study), as the PPV increase with increasing prevalence and NPV decrease with an increase in prevalence. Since two variables were non-normally distributed (left knee down and right knee up at occasion one), Spearman correlation coefficient were calculated in addition to ICC to assess relative reliability. To calculate and assess ICC on non-normally distributed data is a limitation and the ICC results in the present study must therefore be interpreted with some caution.

Finally, a further limitation in this study was the non-existent 3D kinematic gold standard, which might have been even better than the used visual assessment. On the other hand, when a new device is evaluated, and no gold standard is existing, the construct validity is to be used under the assumption that the measurement instrument validly measures the construct to be measured. In this case, visual assessment of knee valgus – which has been found valid against 3D kinematic gold standard in previous studies – was the obvious and most practical choice [10, 64–67].

## Conclusion

Our data show that a novel software program (Qinematic™) based on the Kinect camera V2 has a poor absolute reliability and “substantial” relative reliability when measuring a SLS at the way down, not at the way up. This indicates that Qinematic™ should not be used to monitor individuals on the way up, nor should it be used to monitor individuals over time. Qinematic™ might possibly be of use for screening or following a group of subjects over time. Taking all diagnostic accuracy measures and predictive values into account, the merged results indicate that Qinematic™ can identify subjects with a knee *over* foot position at a cut-off point of 6°, while those who show a knee *medial* to the foot position in addition might need to be assessed with another test, for example a vertical drop jump [68] or similar test that places a higher demand on the knee. In summary, the use of the Qinematic™ net trajectory angle, which estimates the “line of best fit”, cannot be recommended to assess a knee medial to foot position and should be reconsidered.

## Supplementary information

**Additional file 1.** Biomechanical report of Qinematic™. Contains a description of the different variables for the movement screening tests in Qinematic™.

**Additional file 2.** Bland-Altman plots reliability study. Contains Bland-Altman plots for the four variables left knee up, left knee down, right knee up and right knee down.

## Data Availability

The datasets used and/or analysed in the current study are available from the corresponding author on reasonable request.

## Abbreviations

AUC: Area Under the Curve
CI: Confidence Interval
COSMIN: COnsensus-based Standards for the selection of health Measurement Instruments
DLS: Double Leg Squat
IQR: Interquartile Range
MDC: Minimal Detectable Change
NPV: Negative Predictive Value
NTA: Net Trajectory Angle
PA: Percent Agreement
PPV: Positive Predictive Value
QUADAS: The Quality Assessment of Diagnostic Accuracy Studies
ROC: Receiver Operation Characteristic
SE: Standard Error
SEM: Standard Error of Measurement
SD: Standard deviation
SDC: Smallest Detectable Change
SDK: Software Development Kit
SLS: Single Leg Squat
T1: Test occasion one
T2: Test occasion two
