# Supplementary material for: Reliability and validity of a novel Kinect-based software program for measuring a single leg squat
Source: BMC Sports Sci Med Rehabil. 2020 May 11;12:31. doi: 10.1186/s13102-020-00179-8 (PMC7216608; doi:10.1186/s13102-020-00179-8)
Supplement: Supplementary file 1 — Additional file 1. Biomechanical report of Qinematic™. Contains a description of the different variables for the movement screening tests in Qinematic™. [file 13102_2020_179_MOESM1_ESM.pdf]

Overview

Table of content

Table of content	
Standing Posture - Front View	page 2
Standing Posture - Side View	page 3
Standing Balance - Bilateral	page 4
Standing Balance - Unilateral	page 5
Standing Side Bend	page 6
Double Leg Squat	page 7
Single Leg Squat	page 8

General information about scan	
Gender:	Female
Age (years):	35
Weight (kg):	60
Discomfort reported:	No
Recent fall reported:	No
Estimated height (cm):	176
Body mass index (BMI):	19.28
Recorded at (local time):	Tuesday, April 25, 2017 11:56:00 AM
Recorded at (UTC):	Tuesday, April 25, 2017 9:56:00 AM

Movements		
Stand Still - Face View	Attempt #1	Normal mode
Side Bend	Attempt #1	Normal mode
Two Leg Squat	Attempt #2	Normal mode
Balance on Right Leg	Attempt #1	Normal mode
Balance on Left Leg	Attempt #1	Normal mode
Right Leg Squat	Attempt #1	Normal mode
Left Leg Squat	Attempt #1	Normal mode

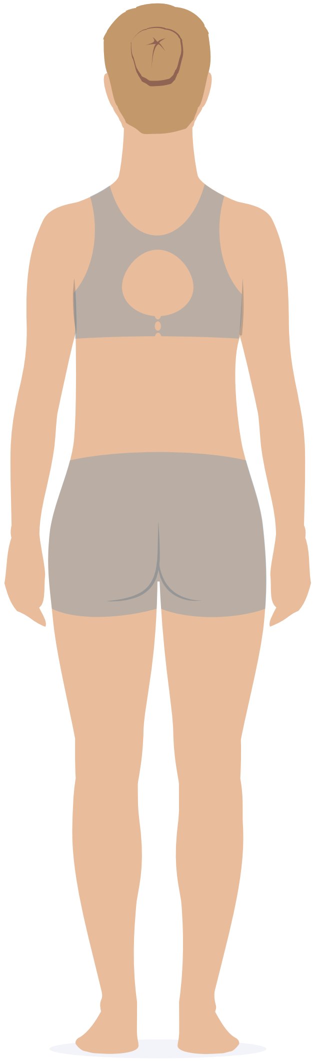

Information in this report should be verified and interpreted by a health professional.
Measures are estimates, calculated from a combination of normative anthropometric data and Qinematic™ Posture Scan 3D test data.

2014-2018 © Qinematic AB info@qinematic.com

Standing Posture - Front View

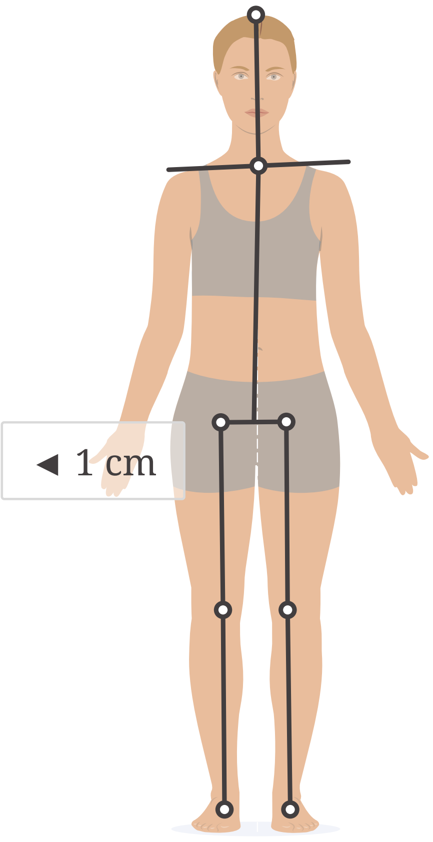

This is the front view of the person whilst standing still.
Vertical alignment is considered zero degrees (deg).

Estimated height loss caused by poor alignment*	
Centimeters	0

*Calculated based on normative data. Measurement error is estimated to be ±1cm

Alignment	Left	Right	Notes
Neck angle of tilt (deg)		1	Relative to frontal axis.
Shoulder angle of tilt (deg)	3		Relative to sagittal axis. Elevated is positive.
Pelvic angle of tilt (deg)	0	0	Relative to sagittal axis. Elevated is positive.

Lateral displacement from midline	Left	Right
Center of head (cm)		1
Center of shoulders (cm)	0	0
Center of body mass (cm)	0	0
Center of pelvis (cm)		1

Critical angles in frontal plane	Left	Right	Notes
Cervicothoracic angle (deg)		2	Between trunk and neck.
Lumbopelvic angle (deg)	2		Trunk relative to pelvis.

Knee angles in frontal plane	Med	Lat
Left knee (deg)	1	
Right knee (deg)	0	0

2014-2018 © Qinematic AB info@qinematic.com

Standing Posture - Side View

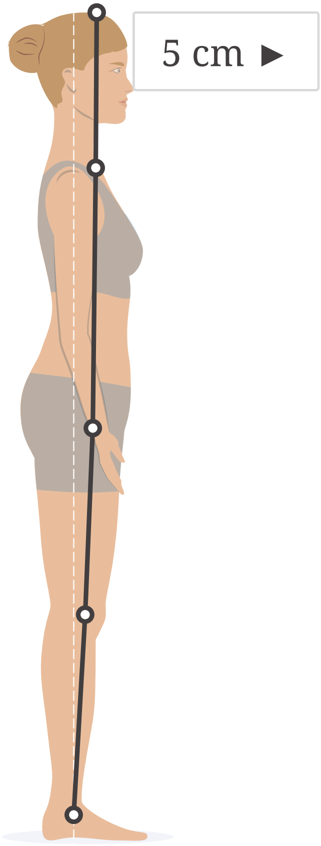

This is the side view with the plumbline (white dashed) and the results from the scan.

Sagittal angle of the neck relative to frontal axis*	
Forward neck angle (deg)	0

*Ventral is positive (+), dorsal is negative (-).

Anterior displacement from plumbline*	
Center of head (cm)	5
Center of shoulders (cm)	5
Center of body mass (cm)	1
Center of pelvis (cm)	4

*Ventral is positive (+), dorsal is negative (-).

Critical angles (forwards/backwards)*		Notes
Cervicothoracic angle (deg)	0	Between trunk and neck.
Lumbopelvic angle (deg)	-2	Between trunk and thigh.

*Ventral is positive (+), dorsal is negative (-).

Knee angles in frontal plane	Flex
Left knee (deg)	0
Right knee (deg)	2

Sagittal angle of the thighs relative to frontal axis*	
Forward angle of the left thigh (deg)	3
Forward angle of the right thigh (deg)	1

*Ventral is positive (+), dorsal is negative (-).

Rotation*	Left	Right
Shoulders axial rotation (deg)	0	0
Pelvic axial rotation (deg)	2	-2

*Ventral is positive (+), dorsal is negative (-).

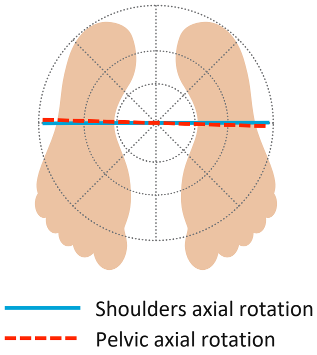

2014-2018 © Qinematic AB info@qinematic.com

Standing Balance - Bilateral

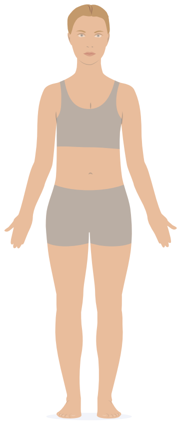

Sway pattern of center of mass in sagittal (anterior/posterior, Ant/Pos) and transversal (mediolateral, Left/Right) plane during 8 seconds of bilateral stance.

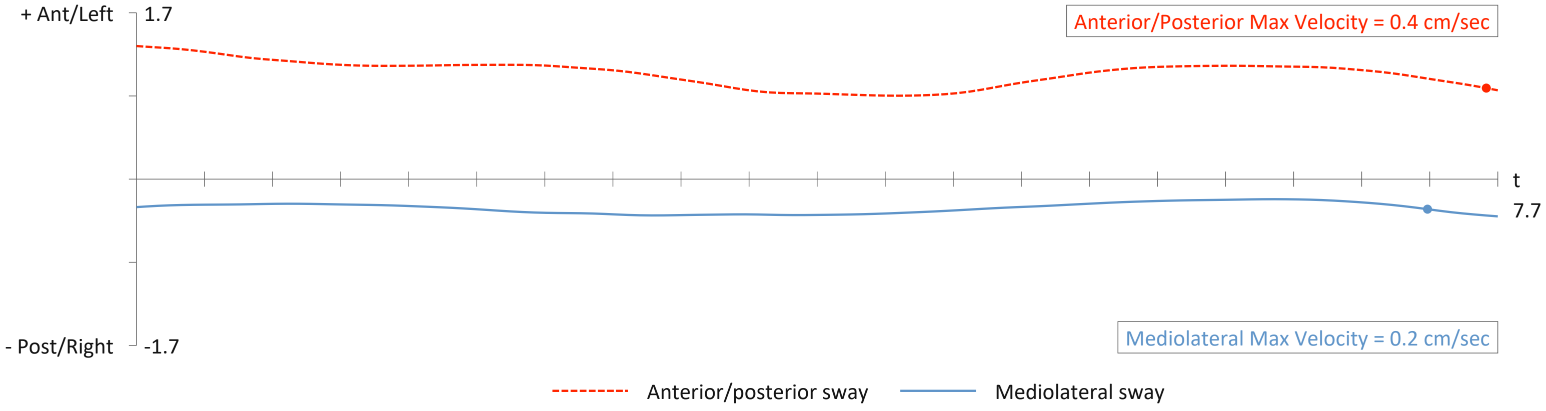

This is the view from cranial of the person in bilateral stance.

The table shows the:
1. Percentage mediolateral displacement (left and right) of the center of mass from the midline.
2. The 'sway area' created by postural sway.

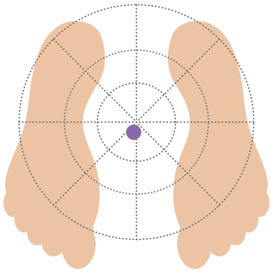

Center of mass
● net position

**Bilateral stance - Eyes Open (Center of mass)**	**Mediolat**	
	**Left**	**Right**
Displacement (%)	48	52
Sway area (cm^2)	0.0	

This footprint view of the body shows the location of the center of mass of the body in relation to the base of support (feet) in bilateral stance.

Information in this report should be verified and interpreted by a health professional.
Measures are estimates, calculated from a combination of normative anthropometric data and Qinematic™ Posture Scan 3D test data.
2014-2018 © Qinematic AB info@qinematic.com

Standing Balance - Unilateral

Sway pattern of center of mass in sagittal (anterior/posterior, Ant/Pos) and transversal (mediolateral, Left/Right) plane during single leg balance.

The movement/time graphs below show the amount of postural sway in anterior (Ant)/posterior (Pos), red and medial (Med)/lateral (Lat), blue direction.
Higher amplitudes correspond with greater movements.

This is the view from cranial of the person in single leg balance.

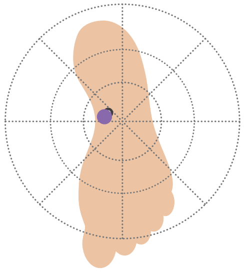

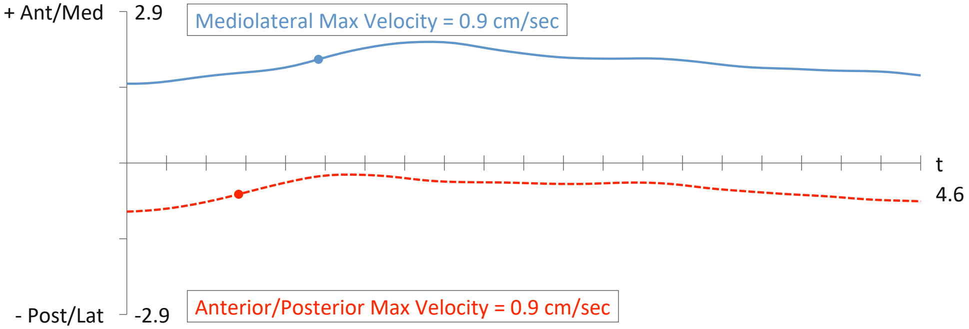

Left leg balance (Center of mass)	Mediolat		Ant/Post	
	Med	Lat	Ant	Post
Displacement (%)	100.0	0.0	0.0	100.0
Sway area (cm²)	0.2			

Center of mass
● net position

Anterior/posterior sway — Mediolateral sway

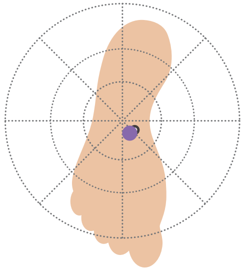

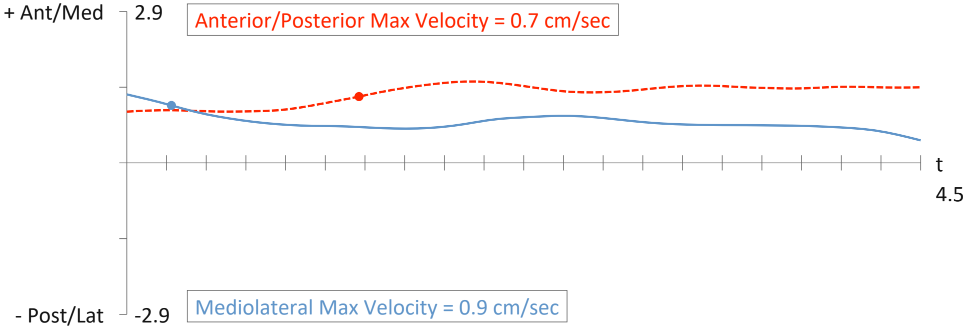

Right leg balance (Center of mass)	Mediolat		Ant/Post	
	Med	Lat	Ant	Post
Displacement (%)	100.0	0.0	100.0	0.0
Sway area (cm²)	0.3			

Information in this report should be verified and interpreted by a health professional.
Measures are estimates, calculated from a combination of normative anthropometric data and Qinematic™ Posture Scan 3D test data.
2014-2018 © Qinematic AB info@qinematic.com

Standing Side Bend

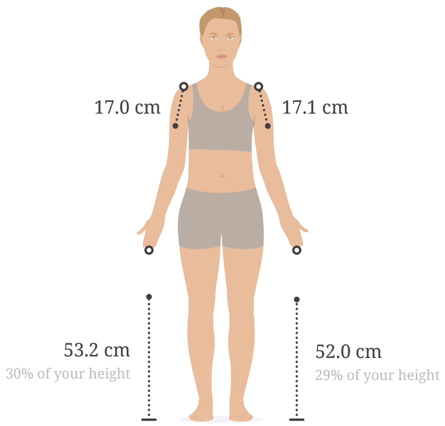

The vertical distance from the floor is used to estimate side reach. Multiple body parts contribute to the movement.

Segmental movements:
The movement/time graphs show the amount of superior/inferior placement and the tabulated data shows the amount of lateral displacement for the hands, chest, shoulders and hips.

A movement is positive when it follows the same direction to the bending side.

Higher amplitudes correspond to greater vertical placement.

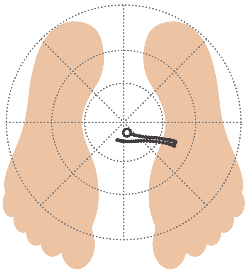

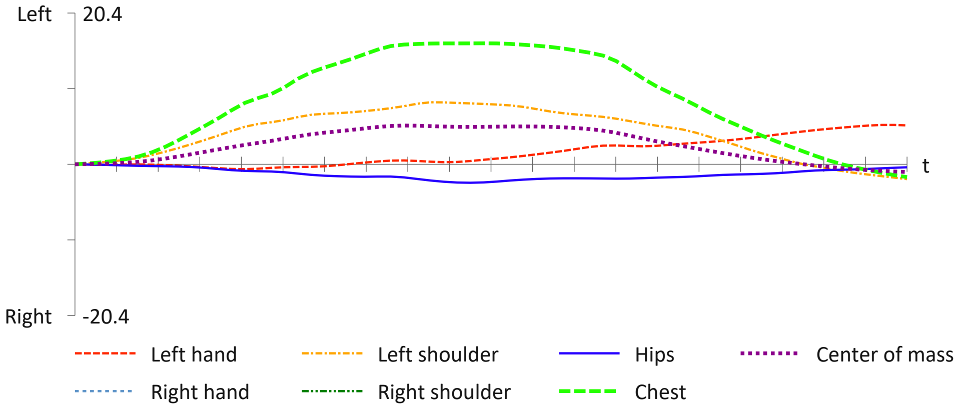

Left Bend	Mediolat		Diff with
	Left	Right	Right Bend
Distance to floor (cm)	52.0		-2%
Weight distribution (%)	80.1	19.9	-
Hips (cm)		2.5	0.4 cm
Chest (cm)	16.3		1%
Left hand (cm)	5.3		-
Left shoulder (cm)	8.4		-9%

Center of mass

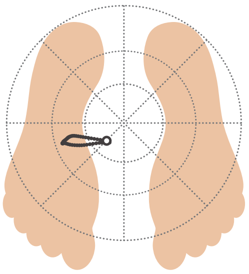

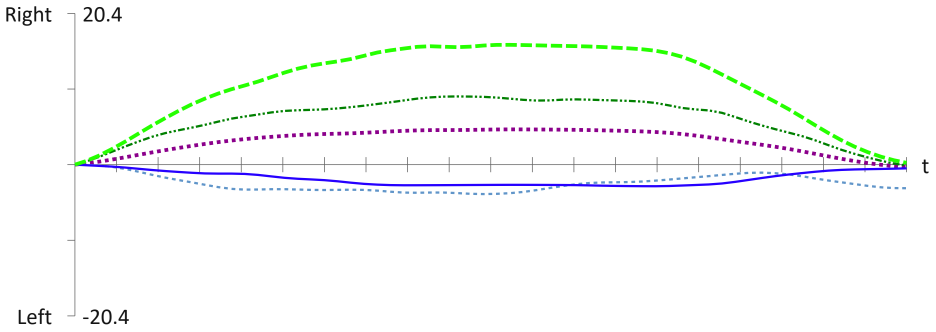

Right Bend	Mediolat		Diff with
	Left	Right	Left Bend
Distance to floor (cm)		53.2	2%
Weight distribution (%)	13.8	86.2	-
Hips (cm)	2.9		-0.4 cm
Chest (cm)		16.2	-1%
Right hand (cm)	4.0		-
Right shoulder (cm)		9.2	10%

Information in this report should be verified and interpreted by a health professional.
Measures are estimates, calculated from a combination of normative anthropometric data and Qinematic™ Posture Scan 3D test data.

2014-2018 © Qinematic AB info@qinematic.com

Double Leg Squat

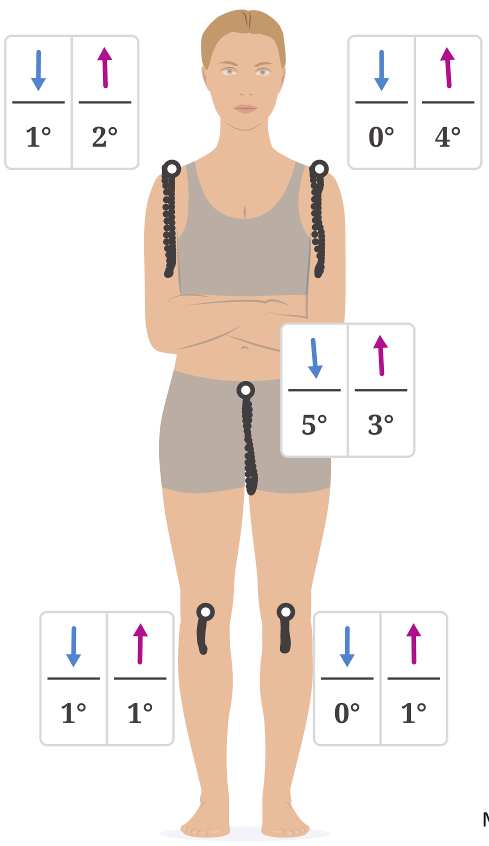

The hips, knees and shoulders are tracked.

The table quantifies the amount of movement in the coronal and transversal planes, as well as the difference between left and right sides for a single squat.
The trajectory angle estimates the line of best fit for the pathway of key body parts. Our own studies indicate that variation between trials is minimal on the way down (eccentric phase) and larger on the way up (concentric phase).

Lateral (Lat) movements are denoted as positive (+).
Medial (Med) movements are denoted as negative (-).
Hip movements to left are denoted as positive (+), hip movements to right are denoted as negative (-).

This is the front view of the person.

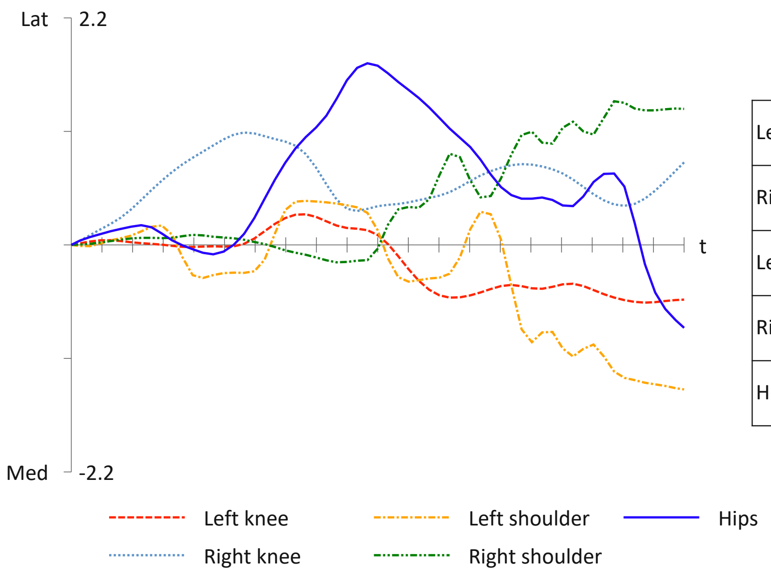

Segment		Vertical Shift		Lateral Shift	Trajectory Angle	
		cm	% diff	cm	deg	diff
Left shoulder	down	21.3	0%	-0.3	0	-4
	up	21.3		-1.1	-4	
Right shoulder	down	21.2	1%	0.9	1	-3
	up	21.5		0.4	2	
Left knee	down	7.5	-3%	-0.5	0	-1
	up	7.7		0.0	-1	
Right knee	down	7.8	4%	0.5	1	0
	up	8.1		0.3	-1	
Hips	down	20.5	0%	1.1	5	2
	up	20.4		-2.0	-3	

Information in this report should be verified and interpreted by a health professional.
Measures are estimates, calculated from a combination of normative anthropometric data and Qinematic™ Posture Scan 3D test data.
2014-2018 © Qinematic AB info@qinematic.com

Single Leg Squat

The hips, knees and shoulders are tracked.

The table quantifies the amount of movement in the coronal and transversal planes, as well as the difference between left and right sides for a single squat.
The trajectory angle estimates the line of best fit for the pathway of key body parts. Our own studies indicate that variation between trials is minimal on the way down (eccentric phase) and larger on the way up (concentric phase).

All references are relative to starting position.

This is the front view of the person.

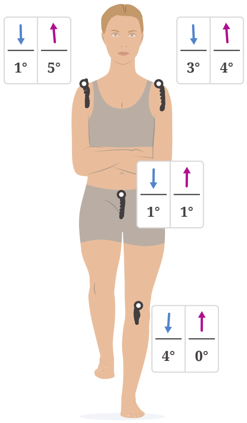

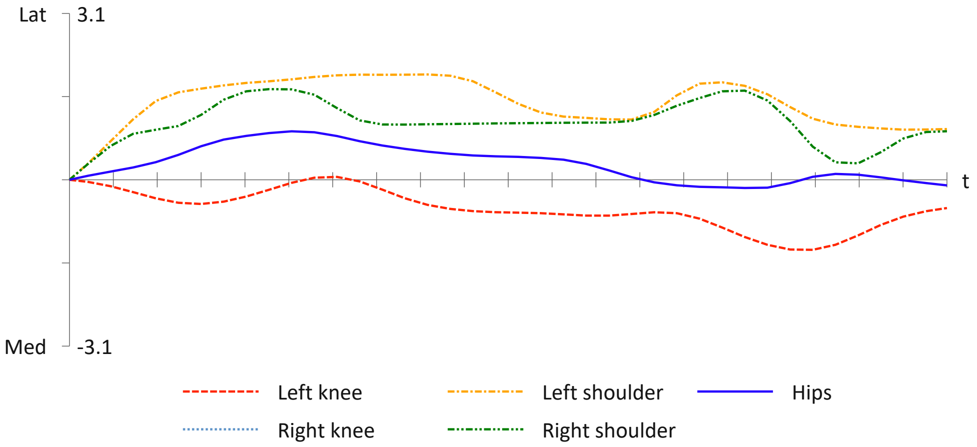

Segment		Vertical Shift		Lateral Shift	Trajectory Angle	
		cm	% diff	cm	deg	diff
Left shoulder	down	10.4	-5%	1.3	3	-1
	up	11.0		-0.3	-4	
Right shoulder	down	9.1	-14%	1.2	1	-4
	up	10.6		-0.3	-5	
Left knee	down	7.0	-8%	-0.6	-4	-4
	up	7.6		0.1	0	
Hips	down	9.2	-2%	0.0	-1	0
	up	9.4		-0.1	1	

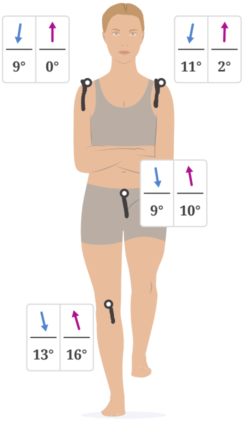

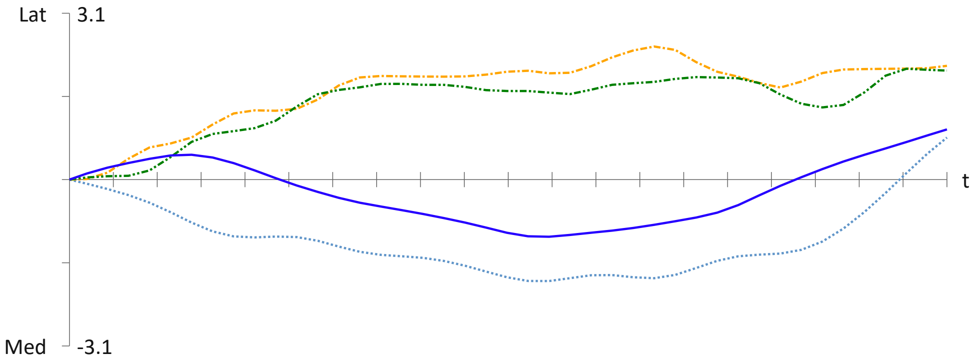

Segment		Vertical Shift		Lateral Shift	Trajectory Angle	
		cm	% diff	cm	deg	diff
Left shoulder	down	9.4	9%	2.3	11	-9
	up	10.4		-0.2	-2	
Right shoulder	down	10.7	5%	1.8	9	-9
	up	11.3		0.3	0	
Right knee	down	7.2	4%	-1.8	-13	-3
	up	7.5		2.5	16	
Hips	down	9.3	5%	-0.9	-9	-1
	up	9.8		1.9	10	

2014-2018 © Qinematic AB info@qinematic.com
