# Supplementary material for: Reliability and validity of a novel Kinect-based software program for measuring a single leg squat
Source: BMC Sports Sci Med Rehabil. 2020 May 11;12:31. doi: 10.1186/s13102-020-00179-8 (PMC7216608; doi:10.1186/s13102-020-00179-8)
Supplement: Supplementary file 2 — Additional file 2. Bland-Altman plots reliability study. Contains Bland-Altman plots for the four variables left knee up, left knee down, right knee up and right knee down. [file 13102_2020_179_MOESM2_ESM.pdf]

Additional file 2-
Bland and Altman plots of reliability study

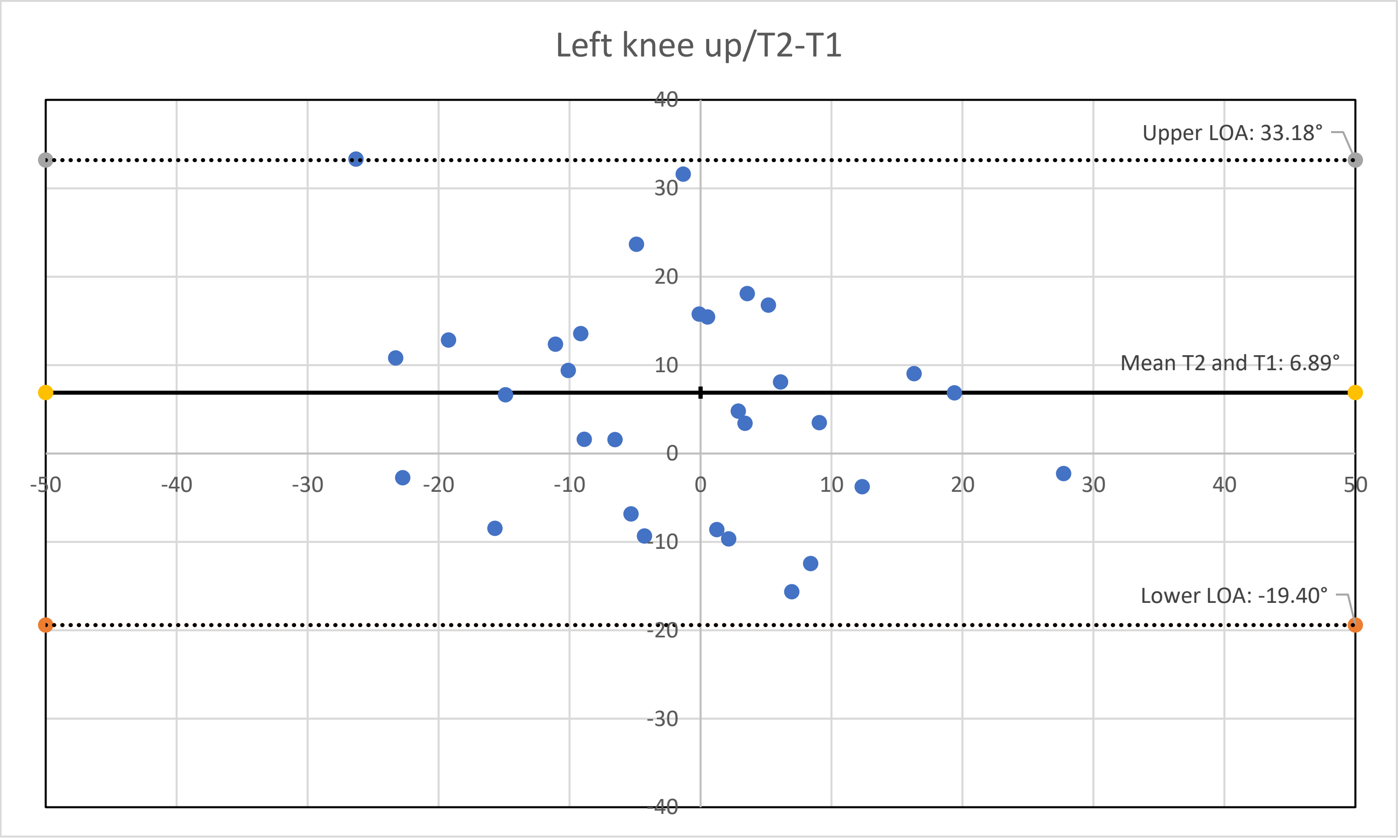

Figure 1. Bland and Altman plots of Knee angles at test occasion one (T1) and two (T2) for the variable left knee up. Differences between T2 and T1 (T2 minus T1), plotted against the mean of T2 and T1. Horizontal lines indicate mean of difference between T2 and T1 and the upper and lower limits of agreement (LOA, 95% limits of agreement, 1,96 ± mean).

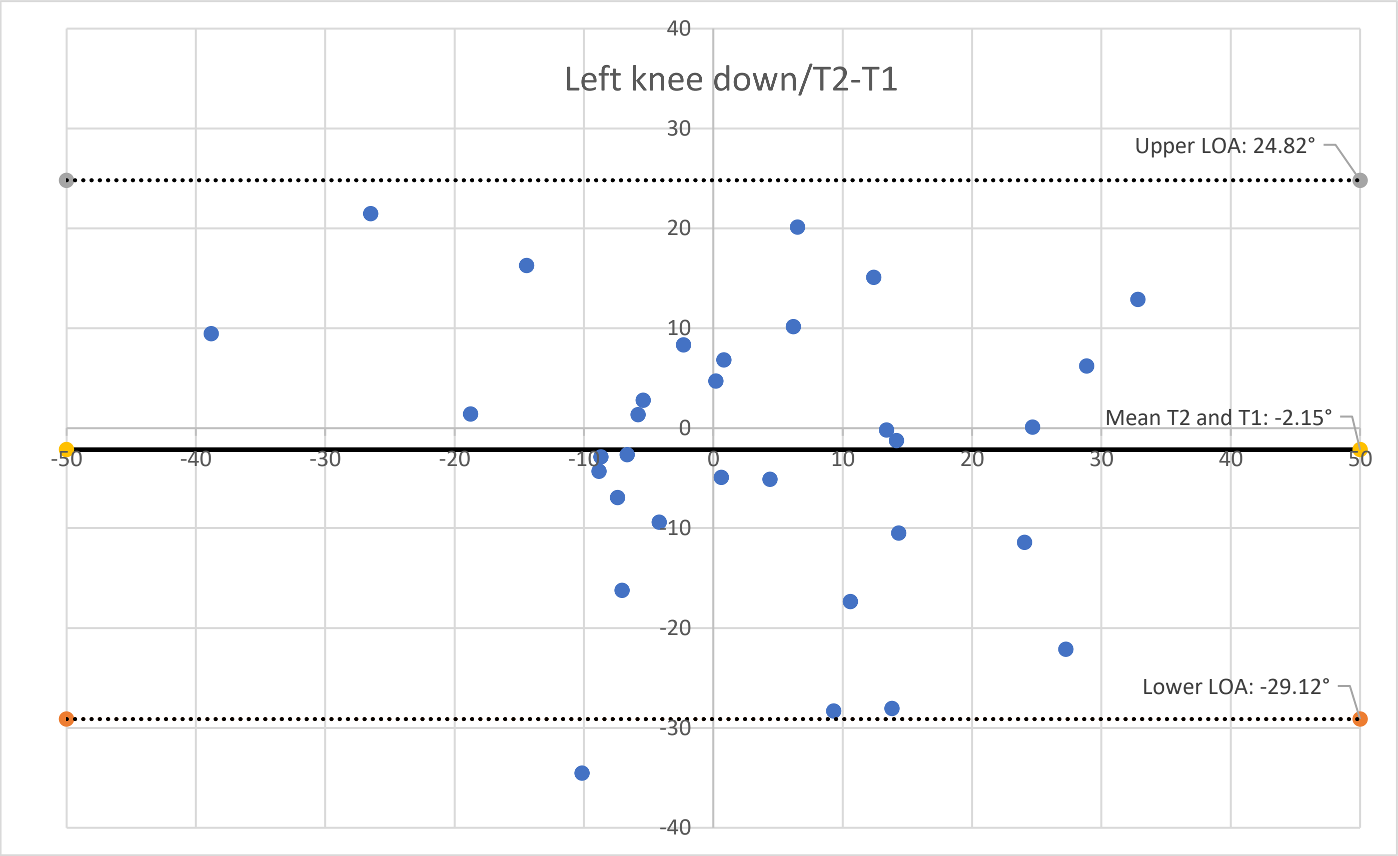

Figure 2. Bland and Altman plots of Knee angles at test occasion one (T1) and two (T2) for the variable left knee down. Differences between T2 and T1 (T2 minus T1), plotted against the mean of T2 and T1. Horizontal lines indicate mean of difference between T2 and T1 and the upper and lower limits of agreement (LOA, 95% limits of agreement, 1,96 ± mean).

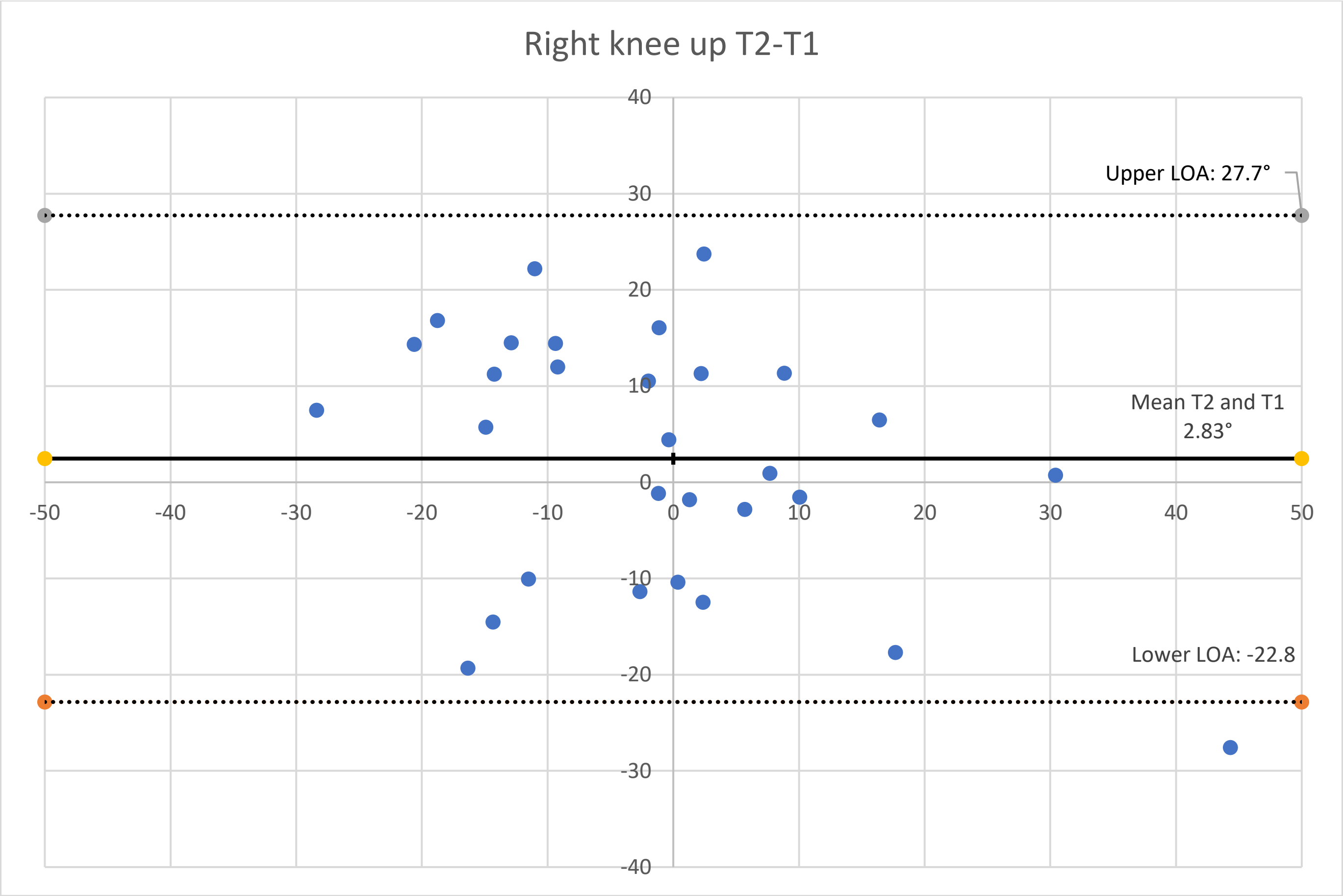

Figure 3. Bland and Altman plots of Knee angles at test occasion one (T1) and two (T2) for the variable right knee up. Differences between T2 and T1 (T2 minus T1), plotted against the mean of T2 and T1. Horizontal lines indicate mean of difference between T2 and T1 and the upper and lower limits of agreement (LOA, 95% limits of agreement, 1,96 ± mean).

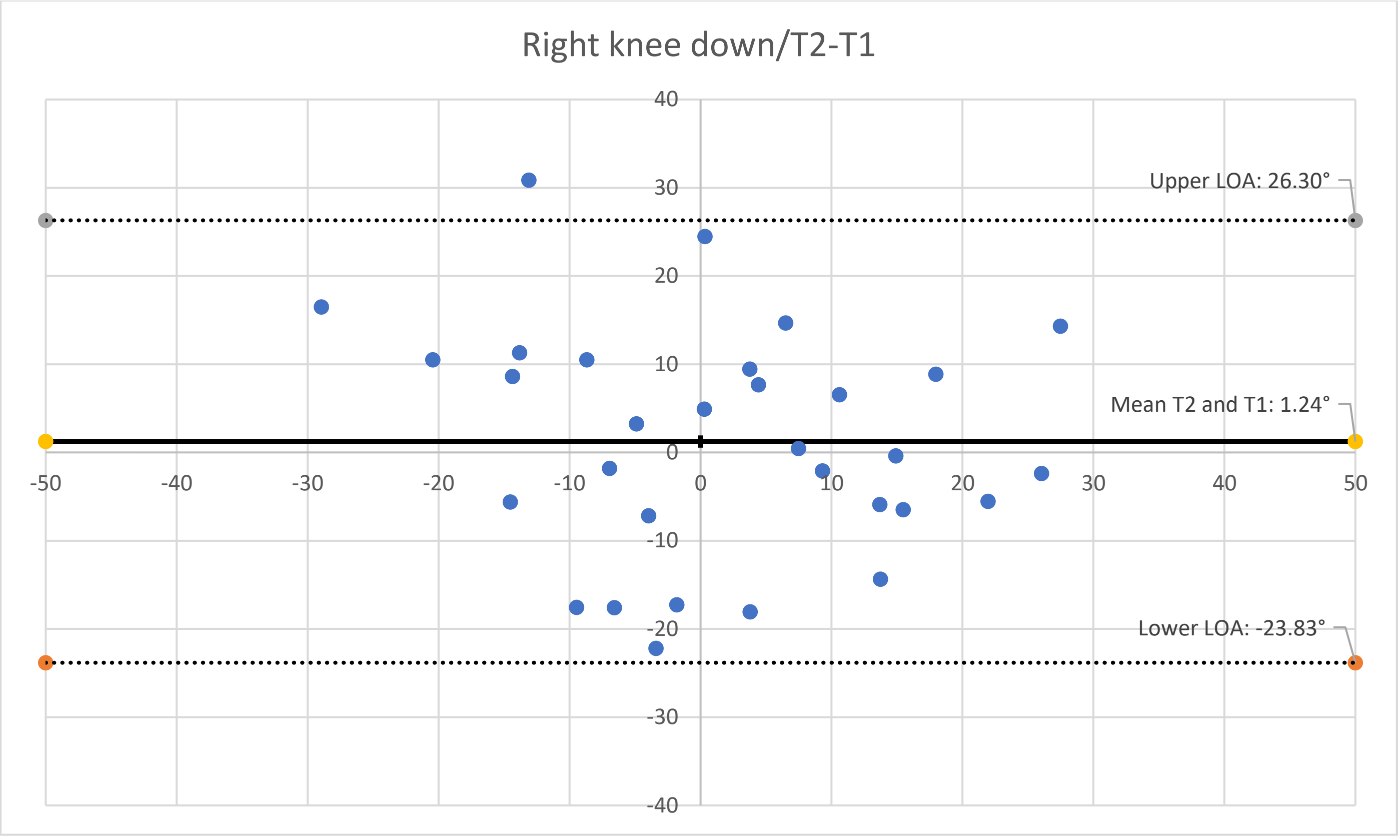

Figure 4. Bland and Altman plots of Knee angles at test occasion one (T1) and two (T2) for the variable right knee down. Differences between T2 and T1 (T2 minus T1), plotted against the mean of T2 and T1. Horizontal lines indicate mean of difference between T2 and T1 and the upper and lower limits of agreement (LOA, 95% limits of agreement, 1,96 ± mean).
